# A Nationwide Study of the Delayed Diagnosis and the Clinical Manifestations of Predominantly Antibody Deficiencies and *CTLA4*-Mediated Immune Dysregulation Syndrome in Greece

**DOI:** 10.3390/medicina60050782

**Published:** 2024-05-08

**Authors:** Androniki Kapousouzi, Fani Kalala, Styliani Sarrou, Evangelia Farmaki, Nikolaos Antonakos, Ioannis Kakkas, Alexandra Kourakli, Vassiliki Labropoulou, Charikleia Kelaidi, Georgia Tsiouma, Maria Dimou, Theodoros P. Vassilakopoulos, Michael Voulgarelis, Ilias Onoufriadis, Eleni Papadimitriou, Sophia Polychronopoulou, Evangelos J. Giamarellos-Bourboulis, Argiris Symeonidis, Christos Hadjichristodoulou, Anastasios E. Germenis, Matthaios Speletas

**Affiliations:** 1 Department of Immunology and Histocompatibility, Faculty of Medicine, University of Thessaly, 41500 Larissa, Greecefkalala@uth.gr (F.K.); ssarrou@uth.gr (S.S.); onoufriadis@gmail.com (I.O.); agermen@med.uth.gr (A.E.G.); 2 Pediatric Immunology and Rheumatology Referral Center, First Department of Pediatrics, Aristotle University of Thessaloniki, 54642 Thessaloniki, Greece; farmakg@auth.gr (E.F.); epapdimitriu@gmail.com (E.P.); 3 4th Department of Internal Medicine, Medical School, National and Kapodistrian University of Athens, 15772 Athens, Greece; n.antonakos@sepsis.gr (N.A.); egiamarel@med.uoa.gr (E.J.G.-B.); 4 Department of Immunology and Histocompatibility Department, “Evaggelismos” General Hospital, 10676 Athens, Greece; ioankakkas@evaggelismos-hosp.gr; 5 Hematology Division, Department of Internal Medicine, University of Patras Medical School-University Hospital, 26504 Patras, Greece; akourakli@hotmail.com (A.K.); vaslabrop@upatras.gr (V.L.); argiris.symeonidis@yahoo.gr (A.S.); 6 Department of Pediatric Hematology-Oncology (T.A.O.), “Aghia Sophia” Children’s Hospital, 11527 Athens, Greece; ch.kelaidi@paidon-agiasofia.gr (C.K.); sophpol@otenet.gr (S.P.); 7 ENT Department, General Hospital of Volos, 38222 Volos, Greece; gtsiouma@ghv.gr; 8 Department of Haematology and Bone Marrow Transplantation, “Laikon” General Hospital, National and Kapodistrian University of Athens, 15772 Athens, Greece; msdimou@med.uoa.gr (M.D.); theopvass@hotmail.com (T.P.V.); 9 Department of Pathophysiology, “Laikon” General Hospital, Medical School, National University of Athens, 11527 Athens, Greece; mvoulgar@med.uoa.gr; 10 Laboratory of Hygiene and Epidemiology, Faculty of Medicine, University of Thessaly, 41222 Larissa, Greece; xhatzi@uth.gr

**Keywords:** predominantly antibody deficiencies, common variable immunodeficiency, IgA deficiency, IgG subclass deficiency, CTLA4, diagnosis

## Abstract

*Background and Objectives*: Predominantly antibody deficiencies (PAD) represent the most common type of primary immunodeficiencies in humans, characterized by a wide variation in disease onset, clinical manifestations, and outcome. Considering that the prevalence of PAD in Greece is unknown, and there is limited knowledge on the clinical and laboratory characteristics of affected patients, we conducted a nationwide study. *Materials and Methods*: 153 patients (male/female: 66/87; median age: 43.0 years; range: 7.0–77.0) diagnosed, and followed-up between August 1979 to September 2023. Furthermore, we classified our cohort into five groups according to their medical history, immunoglobulin levels, and *CTLA4*-mutational status: 123 had common variable immunodeficiency (CVID), 12 patients with “secondary” hypogammaglobulinemia due to a previous B-cell depletion immunotherapy for autoimmune or malignant disease several years ago (median: 9 years, range 6–14) displaying a typical CVID phenotype, 7 with combined IgA and IgG subclass deficiencies, 5 patients with CVID-like disease due to *CTLA4*-mediated immune dysregulation syndrome, and 6 patients with unclassified hypogammaglobulinemia. *Results*: We demonstrated a remarkable delay in PAD diagnosis, several years after the onset of related symptoms (median: 9.0 years, range: 0–43.0). A family history of PAD was only present in 11.8%, with the majority of patients considered sporadic cases. Most patients were diagnosed in the context of a diagnostic work-up for recurrent infections, or recurrent/resistant autoimmune cytopenias. Interestingly, 10 patients (5.6%) had no history of infection, diagnosed due to either recurrent/resistant autoimmunity, or during a work-up of their medical/family history. Remarkable findings included an increased prevalence of lymphoproliferation (60.1%), while 39 patients (25.5%) developed bronchiectasis, and 16 (10.5%) granulomatous disease. Cancer was a common complication in our cohort (25 patients, 16.3%), with B-cell malignancies representing the most common neoplasms (56.7%). *Conclusion*: Our findings indicate the necessity of awareness about PAD and their complications, aiming for early diagnosis and the appropriate management of affected patients.

## 1. Introduction

Inborn errors of immunity (IEI) are a diverse group of rare genetic disorders, affecting almost every aspect of the immune response. Currently, more than 480 different types of IEI have been described [1]. Among IEI, Predominantly Antibody Deficiencies (PAD) are the most common type, affecting both children and adults [2,3]. PAD predisposes individuals to recurrent infections, with a high prevalence of autoimmune manifestations, lymphoproliferation, and atopy, along with a high incidence of cancer, especially lymphomas of B cell origin [1,2,3]. Notably, approximately half of patients with PAD do not exhibit the initial disease manifestations during childhood, but rather during puberty or adolescence [3,4]. With the wide variety of symptoms, this in turn obscures their differential diagnosis leading to a long diagnostic delay, in some cases of up to 10 years or more [3,4]. As a result, PAD patients often present with severe and irreversible complications, including bronchiectasis and/or respiratory insufficiency, resulting in significant socio-economic consequences [2,4,5]. 

Among PAD, common variable immunodeficiency (CVID) is the most prevalent disorder worldwide (approximately 1:25,000 to 1:75,000), although its incidence varies among countries [6,7,8]. CVID patients display hypogammaglobulinemia, leading to recurrent and/or persistent bacterial infections [6,7], along with a wide range of other clinical manifestations (lymphoproliferation, autoimmunity, granulomas formation, etc.), indicative of an extensive immune dysregulation [5,6,9]. The primary criteria for CVID diagnosis include: (a) low serum levels of IgG, IgA and/or IgM, greater than two standard deviations below the normal mean for the age; (b) absent isohemagglutinins and poor responses to vaccines (especially the polysaccharide ones); and (c) an exclusion of other defined causes of hypogammaglobulinemia and/or other types of IEI [7,10]. However, some patients do not meet all the aforementioned CVID criteria, making their diagnosis rather challenging [9]. For instance, this is applicable in the case of combined IgA and IgG subclass deficiencies, since affected patients may display recurrent infections, mainly of the upper respiratory tract, along with non-infectious manifestations, including benign lymphoproliferation and/or autoimmunity [9,11]. 

Notably, the genetic defects leading to overt immunodeficiency are unknown for the majority of PAD patients, including patients with CVID and combined IgA and IgG subclass deficiencies [2]. Interestingly, in some patients with an initial diagnosis of CVID, *CTLA4* mutations may be identified as the causative defects, leading to the reclassification of their condition as an immune dysregulating syndrome [12,13]. These patients display low immunoglobulin levels along with severe autoimmunity, lymphadenopathy, and/or inflammatory bowel disease. Therefore, considering that PAD is a heterogeneous group of disorders, their accurate clinical characterization and further genetic analysis may provide appropriate information to better understand, categorize, and manage the affected patients.

In an effort to unravel part of this complexity, many research groups worldwide conduct national or sub-national registries reporting the clinical phenotypes, laboratory findings, and their correlations with outcomes of PAD patients [4,6,14,15]. In this context, the prevalence of PAD in Greece is unknown, and limited knowledge exists about the clinical and laboratory characteristics of affected patients. This nationwide study reports, for the first time in the literature, data on the clinical presentation, diagnosis delay, and management of Greek PAD patients from 1979 to 2023, and subsequently provides the basis for the first nationwide registry of PAD patients in Greece.

## 2. Materials and Methods

A total of 153 patients (male/female: 66/87; median age: 43.0 years; range: 7.0–77.0) with an initial diagnosis of primary hypogammaglobulinemia (excluding patients with X-linked agammaglobulinemia) between 1979 to 2023 were retrospectively enrolled in the study and their clinical and laboratory data were recorded and analyzed until September 2023. Among them, 143 patients (93.46%) were adults (male/female: 60/83), 9 patients (5.88%; male/female: 6/3) were adolescents and only 1 patient was a child (female, seven years of age), since the majority of participating centers were hospital-level care centers for adult patients (the Immunology Department of University of Thessaly, the Immunology Department of Evangelismos Hospital of Athens, the Department of Internal Medicine of University of Patras, the Department of Internal Medicine of Attikon Hospital of Athens, and the Departments of Pathophysiology and Hematology of Laikon Hospital of Athens). Most patients (140; 91.5%) fulfilled the diagnostic criteria of CVID (as mentioned above) [7,10], while 13 patients (8.5%) displayed combined IgA and IgG subclass deficiencies with a “CVID-like” clinical phenotype. Furthermore, we classified our cohort into groups according to their medical history (previous immune/chemo-therapy or not), immunoglobulin levels, and the *CTLA4* mutational status, as presented in detail below (see Section 3).

The initial diagnostic work-up of patients included a laboratory evaluation of peripheral blood with serology, peripheral blood immunophenotyping (except of the case of patients diagnosed before 2000), radiological imaging, and bone marrow aspiration (for the majority of adult patients) in order to exclude secondary causes of hypogammaglobulinemia. All patients had a follow-up at least every three months in outpatient clinics, or every month for patients receiving intravenous immunoglobulin replacement treatment (IV IgRT). The median follow-up period was 6 years (range: 1–44; Table 1). 

Recorded parameters included demographics, disease symptoms onset (infections or not) that led to medical awareness, age at diagnosis, the duration of the diagnostic delay and clinical symptoms and complications during the follow-up period. Thus, specific attention was given to recorded complications due to infections (nasal polyps, bronchiectasis, chronic obstructive, and/or restrictive respiratory disease), the development and location of granulomatous disease, the presence of benign lymphoproliferation (splenomegaly, reactive lymphadenopathy, intestinal lymphoid infiltrates), as well as associated interventions related to the latter (adenoidectomy, tonsillectomy, splenectomy). We further recorded gastrointestinal manifestations including sprue-like disease, Crohn-like disease, non-specific colitis, and chronic diarrhea due to infections or other causes. The type of autoimmune manifestations and the development of neoplasia were also recorded, with an emphasis on the type of autoimmunity and cancer, along with the time of emergence. 

Our study furthermore focused on the possible consanguinity among ancestors, as well as family medical history of IEI, autoimmune disorders, and/or cancer. Finally, we recorded the type and duration of IgRT, along with the administration of other type of therapy that patients received (i.e., for autoimmune manifestations, cancer, etc.). In terms of laboratory testing, we recorded the quantitative immunoglobulin serum levels (IgG with isotypes, IgA, and IgM) at the time of diagnosis and the immunophenotypic data in peripheral blood if available. Completed forms were imported in a Microsoft Excel 2016 database that is maintained at the Department of Immunology and Histocompatibility, University of Thessaly, Greece.

Written informed consent was obtained from each individual or an accompanying relative, for a few patients whose consent was not legally applicable (e.g., children). The study was designed according to Helsinki II declaration ethics and approved by the ethical committee of the Faculty of Medicine, University of Thessaly, Greece (6/18.3.2015). At the end, descriptive statistics for quantitative variables and frequencies for qualitative variables were calculated through GraphPad Prism Software (San Diego, CA, USA; version 10.1.1).

## 3. Results

### 3.1. Diagnosis, Family History, and the Further Subclassification of PAD Study Patients

As presented in Table 1, the major problem that emerged from recording patients’ data was the remarkable delay of PAD diagnosis, several years after the onset of relevant symptoms (median: 9.0 years, range: 0–43). Most patients were diagnosed with hypogammaglobulinemia in the context of a diagnostic work-up for recurrent infections, or recurrent/resistant autoimmune cytopenias. Moreover, 18 patients were relatives of probands who were initially diagnosed with PAD; thus, a family history of PAD in our cohort was only 11.8%, with the majority of PAD patients considered as sporadic cases. Conversely, medical history records revealed that a great majority of patients (63, 43.1%) had a family history of autoimmune diseases, while 52 patients (33.9%) also had a family history of cancer. 

As mentioned in the Section 2, we further classified our patients into five (5) subgroups according to their medical history, immunoglobulin levels, and *CTLA4* mutational status. In particular, Group A consisted of 123 patients (male/female: 50/73; median age at analysis: 46.0 years; range 7.0–77.0; median age at diagnosis: 37.0 years; range: 4–69) who fulfilled the classical diagnostic criteria of CVID, as mentioned above [7,10] (Table 1). 

In Group B, we included 12 patients (male/female: 9/3; median age at analysis: 39.0 years; range: 26–65; median age at diagnosis: 32.0 years; range: 11–60) (Table 1), who also fulfilled all the aforementioned CVID diagnostic criteria, but displayed a medical history of immunotherapy and/or chemotherapy, due to either a hematological malignancy or autoimmune cytopenias, several years before PAD diagnosis (median: 9 years, range: 6–14). In particular, seven patients with a history of a B cell-malignancy (five with non-Hodgkin lymphomas, and two with acute lymphoblastic leukemia) were treated with regimens containing rituximab, remaining in complete hematological remission for many years before PAD diagnosis. Similarly, five patients had a history of autoimmune cytopenias (three with Evans syndrome, and two with autoimmune thrombocytopenia) who had received immunosuppressant treatment, including rituximab several years before PAD diagnosis; among them, two patients were diagnosed at the relapse of autoimmunity, while the remaining patients were diagnosed during a diagnostic work-up of recurrent infections. Details of clinical and laboratory characteristics of these patients are presented in Supplementary Table S1. 

In Group C, we included 7 patients who displayed combined IgA and IgG4 subclass deficiencies with a clinical phenotype similar to CVID patients (recurrent infections, and/or lymphoproliferation, and/or autoimmune manifestations, and/or enteropathy; and/or granulomas formation; Table 1). Among them, three patients also displayed lower levels of IgG2, three had lower levels of IgG1, and one patient had lower levels of IgG3; however, no patients had IgG1/IgG2/IgG3 deficiency. Their median age at diagnosis was 33.0 years (range: 13–44), and the median age at time of analysis was 45.0 years (range: 19–47) (Table 1).

In Group D, we included 5 patients (male/female: 1/4; median age at diagnosis: 19.0; range: 7–44) who had an initial diagnosis of CVID, but genetic analysis revealed the presence of pathogenic *CTLA4* mutations. The molecular and clinical characteristics of these patients are presented in detail in Table 2, with more clinical data provided in the next subsection.

Finally, in Group E we included 6 patients (male/female: 1/5; median age at diagnosis: 59.0; range: 51–69) who displayed mild to moderate hypogammaglobulinemia with recurrent infections and a negative work-up for secondary immunodeficiencies, but did not fulfill diagnostic criteria of CVID, displaying for example appropriate immune responses after vaccination. Their median age at diagnosis was 59.0 years (range: 51–69), and their median age at time of analysis was 66.0 years (range: 54–71; Table 1).

### 3.2. Clinical Characteristics of the Study Patients

**Figure 1:**
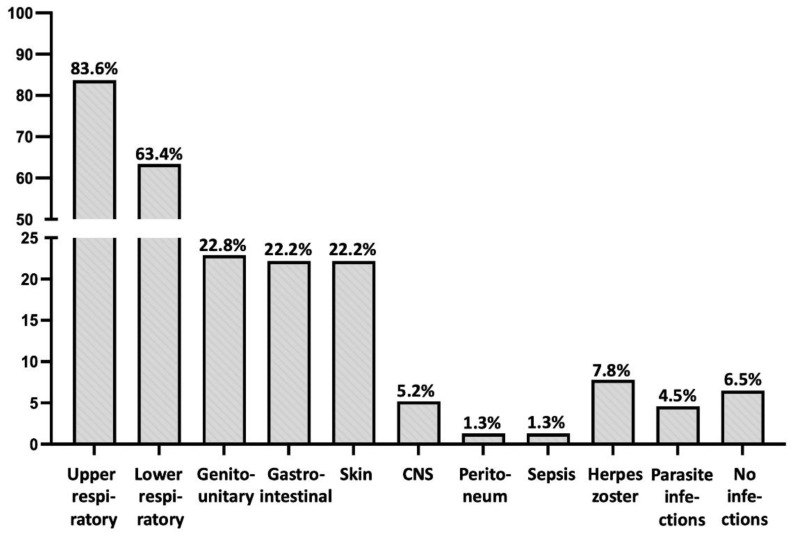
Location and incidence of infections in the patients of the study.

As presented in Table 1, the prevalent clinical problem of PAD patients were recurrent and/or severe infections, with an incidence according to their location detailed in Table 1 and Figure 1. Thus, the most common locations were those of upper and lower respiratory tracts. Central nervous system (CNS) infections (meningitis and/or encephalitis) were less common (8 patients; 5.2%), including a case of brain cysticercosis and another one with severe systemic nocardiosis with brain involvement. Parasitic infections were recorded in seven patients (4.6%), involving gastrointestinal tract (worms, lambdia, ascaris lumbricoides), lungs (strongyloides), and brain (cysticercosis), while 12 patients (7.8%) exhibited at least one attack of herpes zoster. It is noteworthy that patients with combined IgA and IgG subclass deficiencies (Group C) did not display gastrointestinal infections, despite the undetectable IgA levels in their blood (Table 1). 

Interestingly, 55 patients (35.9%) were diagnosed after a work-up for other clinical manifestations, especially of recurrent/resistant autoimmune hematologic manifestations (autoimmune thrombocytopenia, autoimmune hemolytic anemia, Evans syndrome), or unexplained lymphoproliferation (splenomegaly and/or lymphadenopathy). However, most revealed an additional history of recurrent infections (that was considered by the patients as a “normal” condition due to its chronicity), and only 10 patients (6.5%) had no history of infections. The latter group displayed recurrent hematologic or hepatic autoimmunity (6 and two patients, respectively), while two further patients were diagnosed during a conventional work-up. In particular, the first patient displayed a history of acute lymphoblastic leukemia after immunotherapy (being in remission, but without any recovery of immunoglobulin levels more than 7 years after treatment), and the second patient was the brother of a proband with CVID carrying the same pathogenic *IKZF1* (*Ikaros*) mutation (CVID13, OMIM #616873).

**Figure 2:**
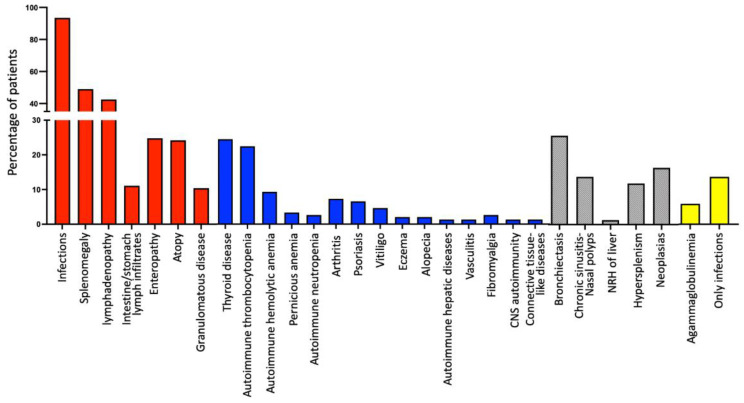
An overview of the clinical manifestations and complications of the patients of the study.

In addition, remarkable findings were the increased prevalence of lymphoproliferation in our cohort of patients, regardless of their subclassification (Table 1, Figure 2). Moreover, 16 patients (10.5%) developed granulomatous disease, 13 patients in Group A and 1 patient in each of the other groups, with the exception of Group E (Table 1). Lungs were the most frequent location (10 patients; 62.5%), but granulomas were also found in the liver (5 patients; 31.2%), skin (two patients; 12.5%), spleen (one patient; 6.3%), and lymph nodes (one patient; 6.3%). Only three CVID patients exhibited multisystemic granulomatosis, with granulomas being formed in more than one location apart from liver (lymph nodes, spleen, and lungs, respectively). It should be noted that two of these 16 patients (12.5%) were initially misdiagnosed with sarcoidosis, receiving corticosteroid treatment for one and three years, respectively, resulting in severe lower respiratory infections in one case and severe lung parasitosis in the other, since they were not receiving IgRT. 

Interestingly, patients with *CTLA4* defects (all located into exon 2 of *CTLA4* gene) displayed a variable clinical phenotype at diagnosis, as presented in detail in Table 2. Thus, four out of five patients were diagnosed during a work-up of recurrent infections, while one of these patients also displayed a massive splenomegaly. The fifth patient was diagnosed after a traffic accident, when a diffuse lymphoid hyperplasia, splenomegaly and hypogammaglobulinemia—found incidentally—initially aroused suspicion of a lymphoid malignancy. After a comprehensive work-up and worsening hypogammaglobulinemia over time, the patient was diagnosed with PAD, while the final diagnosis of a *CTLA4* defect and the immune dysregulating syndrome were made postmortem. It is worth noting that two patients with *CTLA4* defects displayed no autoimmune manifestations, while the remaining three suffered from autoimmune thyroiditis, and one also suffered from pernicious anemia (Table 2). Clinical and laboratory data for three of five patients with *CTLA4* defects, along with detailed functional studies for one of them, were also presented by us in previous manuscripts [16,17]. 

Only 21 patients (13.7%, male/female: 11/12; median age of disease onset: 20 years, range: 1–48; median age at diagnosis: 37.0 years, range: 4–53) displayed only infections, without any other manifestation of PAD (including lymphoproliferation, autoimmunity, atopy, enteropathy, or granulomatous disease) (Figure 2); this included 16 patients from Group A, 2 patients from Group B and 2 patients from Group E (Figure 2). All these patients were alive in September 2023, with a median follow-up period of 5 years (range: 5–21). 

The majority of our PAD patients (142 of 153, 92.8% in total; 118 patients, 95.3% of Group A; 10 patients, 83.3% of Group B; four patients, 57.1% of Group C; 5 patients, 100.0% of Group D; 5 patients, 83.3% of Group E) systematically received IgRT, with most of them subcutaneous IgRT (108 patients; 76.1%). As mentioned above, 11 patients did not receive IgRT due to an absence of infections, especially in cases with moderate IgG hypogammaglobulinemia (levels above 400 mg/dL); however, two patients denied IgRT (despite recommendations), receiving only antibiotic treatment during infections.

### 3.3. Laboratory Findings of the Study Patients

An overview of immunoglobulin levels and immunophenotyping data in peripheral blood at diagnosis is presented in detail in Table 3. A notable finding was the almost complete absence of immunoglobulins (IgG levels undetectable or below 12 mg/dL, along with rather undetectable IgM and IgA levels, namely a presence of agammaglobulinemia) in the peripheral blood of 9 PAD patients (5.9%); among them, 8 patients belonged to Group A and 1 patient to Group B. 

In addition, four male patients (2.6% of the total) with an onset of disease in adulthood (mean age of onset: 31 years, range: 18–44) exhibited undetectable levels of IgG and IgA at diagnosis, along with IgM levels into the normal range. Molecular analyses for the known causatives of hyper-IgM syndromes were negative, while a patient carried the pathogenic CVID mutation *TNFRSF13B*-p.C104R (rs34557412) in heterozygous state. 

Further statistical analyses revealed no significant differences in all immunoglobulin subtypes (including IgG subclasses) between patients of groups A and B (*p* > 0.05 in all cases). Patients with *CTL4*-mediated immune dysregulation syndrome (Group D) displayed significantly higher IgG, IgA and IgG1 levels compared to patients of group A (*p* = 0.027, *p* = 0.001 and *p* = 0.043, respectively). Moreover, patients with unclassified hypogammaglobulinemia (Group E) displayed only significantly higher IgA levels compared to patients of Group A (*p* = 0.042). At the end, patients with combined IgA and IgG4 subclass deficiency (group C) displayed significantly higher levels of IgG (*p* < 0.001), IgM (*p* = 0.036), IgG1 (*p* < 0.001), IgG2 (*p* = 0.011), and IgG3 levels (*p* < 0.001) compared to patients in Group A. Considering lymphocyte subpopulations at diagnosis, the only significant difference was the higher levels of switched-memory B cells in peripheral blood of patients with unclassified hypogammaglobulinemia (Group E) compared to patients with CVID (Group A) and *CTLA4*-mediated dysregulation syndrome (Group D) (*p* = 0.014 and *p* = 0.024, respectively; Table 3).

### 3.4. Complications in Our Cohort of Patients

The most common complication was the development of severe chronic respiratory disease, either chronic bronchiectasis, or chronic obstructive and/or chronic restrictive pulmonary disease, or chronic sinusitis with nasal polyps (Table 1). Bronchiectasis was present in 39 patients (25.5%) being one of the prevalent complications in Group A patients (37; 30.0%), including an individual that underwent lobectomy due to extensive bronchiectasis, leading eventually to defective lung function. Conversely, only one patient (14.3%) in Group C and one in Group E (16.7%) developed bronchiectasis, while no patient from Groups B and D developed this complication (Table 1). 

Moreover, two patients with CVID (Group A) developed nodular regenerative hyperplasia (NRH) of the liver, which was confirmed by histology (Table 1); one of them died due to its complications. 

Eighteen patients (11.8%) underwent splenectomy either at diagnosis in the context of differential diagnosis of a potential lymphoproliferative neoplasm (5 patients, 27.8%), or during the follow-up period for therapeutic reasons (13 patients, 72.2%), including refractory autoimmune hematologic manifestations (9 patients with refractory autoimmune thrombocytopenia or Evans syndrome), or severe hypersplenism (4 patients) leading to spontaneous spleen rupture in one patient. 

**Figure 3:**
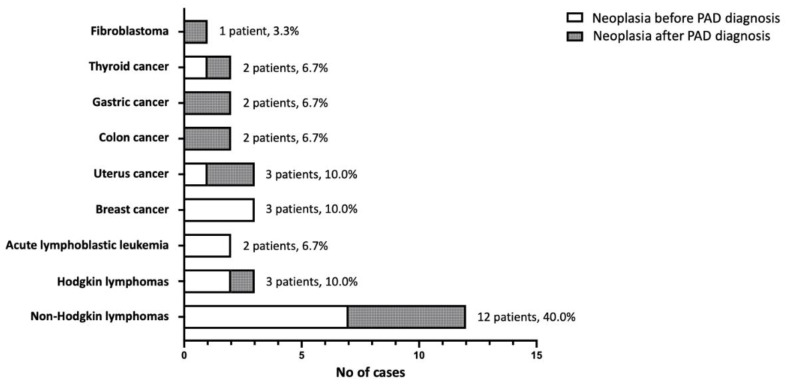
The type of malignancies developed in the patients of the study, before and after PAD diagnosis.

Furthermore, neoplasia was a common complication in our cohort (25 patients, 16.3%). However, considering that several patients had developed cancer many years before PAD diagnosis (Table 1), it is worth noting that five patients displayed more than one type of neoplasia; in particular, two patients with an initial diagnosis of Hodgkin lymphoma relapsed with Non-Hodgkin lymphomas (Burkitt and marginal zone, respectively), a patient with an initial diagnosis of Burkitt lymphoma relapsed with Hodgkin lymphoma, a patient with marginal zone lymphoma relapsed with a diffuse large B cell lymphoma (DLBCL), and another patient developed both cervical and colon cancer. Nevertheless, in the majority of cases there was a past history of recurrent infections, but either no test for immunoglobulin levels had been performed appropriately, or an established hypogammaglobulinemia was not considered (as in a patient diagnosed with severe hypogammaglobulinemia and splenomegaly/splenectomy with granulomas formation 7 years before developing Hodgkin disease and a concomitant PAD diagnosis in a tertiary University Hospital). As presented in Figure 3, hematological malignancies of B-cell origin represent the most common neoplasms developed in our cohort (17 out of 30 cases, 56.7%). Complications of malignancies were the cause of death in three patients (12.0%).

In the end, 14 patients (9.2%) died during the follow-up period (Table 1), with the exception of one patient who passed away due to an automobile accident. In all other cases, the cause of death was a PAD complication (severe infections in four patients—in two with CNS involvement; COVID-19 in two patients; autoimmune complications in two patients; pulmonary hypertension in one patient; NRH complications in one patient; neoplasia in three patients, two due to Non-Hodgkin lymphomas, and one due to colon cancer).

## 4. Discussion

Our study represents the first nationwide study recording the clinical and laboratory manifestations of PAD patients in Greece. It is clear that the major problem in our cohort was a remarkable delay of the precise diagnosis. Thus, many patients experienced incorrect recognition of their condition for several years, resulting in a worsening of their medical status when the final diagnosis was performed. This is a very common phenomenon worldwide, since PAD patients remain largely mis- and/or under-diagnosed, a fact substantially affecting patients’ prognosis and disease outcome. In this context, several groups have conducted national, sub-national, or multi-national studies collecting data in an attempt to record clinical and laboratory characteristics of PAD, while also raising awareness about the importance of timely diagnosis [4,8,11,15]. 

In our study, most PAD patients suffer from CVID (Group A, Table 1). As mentioned above, the delay of disease diagnosis was higher in our cohort compared to other European countries (median: 9.0 vs. 2.1 and 1.8 years in the Netherlands and Poland, respectively), or to the entire European continent based on ESID data (median 4.0 years) [4]. However, in other countries the diagnostic delay was higher, as reported in studies from Mexico and Turkey (median: 12.5 and 14.0 years, respectively) [18,19,20]. Clearly, these differences are due to patients’ selection criteria and data. The majority of our patients were adults (Table 1) without early disease onset in childhood. It should be considered that when the disease manifests in early life, there is an increased suspicion of genetic disorders by pediatricians, with attention for adequate disease diagnosis and patients’ management. Obviously, an awareness of early disease recognition and diagnosis in adulthood is also necessary, as delayed diagnosis is usually complicated by severe and irreversible manifestations (for example bronchiectasis, chronic obstructive pulmonary disease, respiratory insufficiency, etc.) [2,3,4,5].

One of the most interesting study findings was the demonstration of CVID clinical and laboratory phenotypes in patients of Group B, namely in patients initially diagnosed with hematological or autoimmune diseases, receiving immunosuppression therapy against B cells (rituximab in the great majority of cases, Supplementary Table S1) and developing a sustained immunodeficiency that never recovered several years after initial diagnosis (Supplementary Table S1). Actually, we consider these patients as suffering from overt CVID, as they displayed common clinical and laboratory findings, as presented in Table 1, Table 3 and Supplementary Table S1, but we distinguished them in the presentation of their data, due to their previous history of malignancy or autoimmunity and the previous administration of rituximab. It is known and expected that in many patients, the administration of rituximab results in a reduction of B cells and immunoglobulin levels, but these effects are transient and recovered over time [1,21]. However, as already described by other groups, rituximab may represent a triggering factor for the emergence of an overt disease in individuals with a genetic predisposition for immunodeficiency [22,23,24]. Therefore, it is necessary to consider this uncommon “complication” in patients receiving rituximab or similar agents for appropriate monitoring, early diagnosis, and proper management. In this context, we further suggest that these patients may represent a distinct group of IEI/CVID, and this point should be clarified after the integration of their genetic and/or epigenetic studies.

Consistent with other studies in the literature [18,20], recurrent respiratory infections were the most frequent infection type in our cohort, and the most common symptom at disease onset (Table 1). It has been established that chronic inflammatory stimulation due to recurrent respiratory infections leads to structural damages in the respiratory system. This presents as nasal polyps, bronchiectasis, chronic obstructive, and/or restrictive respiratory disease, resulting in the reduction in respiratory capacity. Therefore, similar to previous studies [8,25,26], we observed a rather high prevalence of bronchiectasis in our cohort that was more prevalent in CVID patients (Group A; 30.0%), while it was absent in patients with *CLTA4* mutations (Group D). Unexpectedly, bronchiectasis formation was also absent in Group B patients; we suggest this finding (in Groups B and D) may be due to a rather shorter time between disease onset and patient enrollment, compared to other groups (Table 1). However, these findings and their potential explanation would be confirmed in further studies.

Similarly to other studies [4,14,15,18,27], we demonstrated that autoimmune thyroid disease, autoimmune thrombocytopenia, and autoimmune hemolytic anemia were the most prevalent autoimmune conditions in PAD patients. As mentioned in the Section 3, it should be noted that recurrent and/or resistant autoimmune manifestations were the reason behind disease diagnosis in several patients (55, 35.9%), despite the presence of a past history of infections. Moreover, 10 of 153 patients in our cohort (6.5%) had no history of infections; 8 patients displayed only autoimmune manifestations (2 patients with recurrent/resistant autoimmune thrombocytopenia, 1 patient with recurrent autoimmune hemolytic anemia, 3 patients with Evans syndrome and 2 patients with hepatic autoimmunity—Table 1). Obviously, the presence of recurrent and/or resistant autoimmune manifestations—especially in the context of hematologic autoimmunity—should increasingly raise both suspicion of an underlying immunodeficiency and the need for appropriate diagnostic work-up. 

As detailed in the Section 3 and Table 1, lymphoproliferation (splenomegaly—defined as spleen enlargement on clinical or laboratory examination, lymphadenopathy, and intestinal lymphoid infiltrates—established by endoscopy and confirmed in intestine biopsies) was quite common in our cohort (60.8%). Specifically, splenomegaly rates were higher in our study (52.4%) compared to the studies conducted by Ho and Cunningham (20.9%) [28], Chapel et al. [26] (30%), and Wehr et al. (40.5%) [29], but lower compared to a study by Graziano et al. (65%) [30]. Moreover, the prevalence of lymphadenopathy in our cohort (44.1%) was also higher compared to studies by Wehr et al. (26.2%) [29] and Chapel et al. (30%) [26], but closer to prevalence reported by Musabak et al. (48.4%) [19]. Notable differences in the incidence of lymphoproliferation rates between the aforementioned studies likely correspond to the disease heterogeneity worldwide. Further studies incorporating molecular data through genetic analysis of enrolled patients may clarify the association between clinical presentation of PAD and the genetic background of affected patients. 

In this study, we also reported on patients with an initial diagnosis of CVID who were eventually categorized as suffering from immune dysregulation syndrome due to their *CTLA4* mutational status (Table 1 and Table 2). Heterozygous *CTLA4* mutations have been implicated in the emergence of a complex immune dysregulation syndrome with features of autoinflammation, autoimmunity and immunodeficiency [12,13,31,32], as well as an increased risk of cancer development [32]. Schwab et al. characterized a worldwide cohort of 133 *CTLA4* mutation carriers concerning their clinical and laboratory features. Similar to our study, they observed that most mutations were located in exon 2 of the *CTLA4* gene, encoding the ligand binding and dimerization domains [32]. However, a rather incomplete clinical penetrance has been reported by us and others for the *CTLA4* haploinsufficiency [13,16,17,31], raising the question of additional genetic lesions that may contribute to the emergence and clinical phenotype of the disease. In this context, it is also interesting that we describe a new patient with a *CTLA4* defect (#116, Table 2), without any sign of autoimmunity or autoinflammation, displaying only recurrent infections and splenomegaly. This suggests that *CTLA4* molecular analysis is necessary in all patients with PAD. 

One of our study limitations is the fact that we did not have molecular data for the entire cohort of patients (with the exception of the molecular analysis of *CTLA4* gene for all and *TNFRSF13B/TACI* for the great majority of the enrolled patients [33]. Consequently, we have not performed association studies of the genetic background of affected individuals with the phenotype of their disease. However, the principal purpose of our nationwide study was to initially select the demographic, clinical, and laboratory data from Greek patients with PAD, in order to proceed with the organization of a national registry which could facilitate collaboration between centers and promote the molecular analysis of patients and their families. 

## 5. Conclusions

In conclusion, this is the first nationwide study of PAD patients in Greece. We demonstrated a remarkable delay of disease diagnosis, while we also observed a variable disease phenotype, with a proportion of patients developing PAD several years after an initial diagnosis of autoimmunity or malignancy, and their appropriate therapy. Clearly, these findings indicate the necessity of awareness about PAD and their complications, aiming for early diagnosis and the appropriate management of affected patients.

## Figures and Tables

**Table 1:** Overview of the demographic and clinical characteristics of the patients during the entire follow-up period of the study.

	Total	Group A (CVID)	Group B ^∇^ (“Secondary” Hypogamma)	Group C (c.IgAD and IgGsD)	Group D (*CLT4* Deficiency)	Group E (Unclassified Hypogamma)
No	153	123	12	7	5	6
Sex (male/female)	66/87	50/73	9/3	5/2	1/4	1/5
Age of analysis (years; median, range)	43.0 (7.0–77.0)	44.0 (7.0–77.0)	35.0 (22.0–61.0)	42.0 (15.0–45.0)	28.0 (21.0–48.0)	66.0 (54.0–71.0)
Age of diagnosis (years; median, range)	37.0 (4.0–69.0)	37.0 (4.0–69.0)	32.0 (11.0–60.0)	33.0 (13.0–44.0)	19.0 (7.0–44.0)	58.0 (46.0–69.0)
Delay of diagnosis (years; median, range)	9.0 (0–43.0)	9.0 (0.0–43.0)	6.5 (1.0–17.0)	12.0 (0.0–36.0)	4.0 (0.0–25.0)	7.5 (2.0–33.0)
Duration of follow-up (years; median, range)	6.0 (1.0–44.0)	7.0 (1.0–44.0)	3.0 (1.0–23.0)	4.0 (3.0–11.0)	7.0 (3.0–44.0)	6.5 (1.0–18.0)
**Clinical manifestations**						
Infections (no, %)	143 (93.5)	116 (94.3)	9 (75.0)	7 (100.0)	5 (100.0)	6 (100.0)
Upper respiratory (no, %)	128 (83.7)	106 (86.1)	8 (66.7)	6 (85.7)	4 (80.0)	4 (66.7)
Lower respiratory (no, %)	97 (63.4)	83 (67.5)	5 (41.7)	3 (42.9)	3 (60.0)	3 (50.0)
Genitourinary (no, %)	35 (22.9)	31 (25.2)	1 (8.3)	1 (14.3)	0 (0)	2 (33.3)
Gastrointestinal (no, %)	34 (22.2)	28 (22.7)	1 (8.3)	0 (0)	2 (40.0)	3 (50.0)
Skin (no, %)	34 (22.2)	30 (24.4)	2 (16.7)	0 (0)	1 (20.0)	1 (16.7)
CNS (no, %)	8 (5.2)	7 (5.7)	0 (0)	0 (0)	1 (20.0)	0 (0)
Peritonitis (no, %)	2 (1.3)	1 (8.1)	0 (0)	0 (0)	1 (20.0)	0 (0)
Sepsis (no, %)	2 (1.3)	2 (1.6)	0 (0)	0 (0)	0 (0)	0 (0)
Parasite infections (no, %)	7 (4.6)	7 (5.7)	0 (0)	0 (0)	0 (0)	0 (0)
Herpes zoster (no, %)	12 (7.8)	11 (8.9)	0 (0)	0 (0)	0 (0)	1 (16.7)
Lymphoproliferation (no, %)	92 (60.1)	79 (64.2)	5 (41.7)	3 (42.9)	3 (60.0)	2 (33.3)
Splenomegaly (no, %)	75 (49.0)	62 (50.4)	5 (41.7)	3 (42.9)	3 (60.0)	2 (33.3)
Lymphadenopathy (no, %)	65 (42.5)	56 (45.5)	5 (41.7)	2 (28.6)	2 (40.0)	0 (0)
Intestine infiltrates (no, %)	17 (11.1)	15 (12.2)	0 (0)	1 (14.3)	1 (20.0)	0 (0)
Autoimmunity (no, %)	87 (56.9)	69 (56.1)	7 (58.3)	6 (85.7)	3 (60.0)	2 (33.3)
ITP or/and AHA (no, %)	38 (24.8)	29 (23.6)	5 (41.7)	3 (42.9)	0 (0)	1 (16.7)
Thyroid disease (no, %) ^	37 (24.2)	28 (22.8)	1 (8.3)	3 (42.9)	3 (60.0)	2 (33.3)
Arthritis (no, %) ^^	11 (7.2)	11 (8.9)	0 (0)	0 (0)	0 (0)	0 (0)
Psoriasis (no, %)	10 (6.5)	8 (6.5)	2 (16.7)	0 (0)	0 (0)	0 (0)
Others (no, %) ^#^	35 (22.9)	28 (22.8)	2 (16.7)	2 (28.6)	2 (40.0)	1 (16.7)
Granulomatosis (no, %)	16 (10.4)	13 (10.6)	1 (8.3)	1 (14.3)	1 (20.0)	0 (0)
Enteropathy (no, %) ^##^	38 (24.8)	29 (23.6)	1 (8.3)	2 (28.6)	3 (60.0)	3 (50.0)
Atopy and/or drug allergy (no, %) ^###^	37 (24.2)	32 (26.0)	1 (8.3)	1 (14.3)	2 (40.0)	1 (16.7)
**Complications of the disease**						
Bronchiectasis (no, %)	39 (25.5)	37 (30.0)	0 (0)	1 (14.3)	0 (0)	1 (16.7)
COPD (no, %)	19 (12.4)	15 (12.2)	0 (0)	2 (28.6)	0 (0)	2 (33.3)
CRPD (no, %)	25 (16.3)	24 (19.5)	1 (8.3)	0 (0)	0 (0)	0 (0)
Chronic sinusitis (no, %) *	21 (13.7)	17 (13.8)	2 (16.7)	1 (14.3)	0 (0)	1 (16.7)
NRH (no, %)	2 (1.3)	2 (1.6)	0 (0)	0 (0)	0 (0)	0 (0)
Hypersplenism (no, %) **	18 (11.8)	15 (12.2)	2 (16.7)	0 (0)	0 (0)	0 (0)
**Neoplasia-total (no, %)**	25 (16.3)	16 (13.0)	7 (58.3)	0 (0)	1 (20.0)	1 (16.7)
Neoplasia after PAD diagnosis (no, %)	11 (7.2)	10 (8.1)	0 (0)	0 (0)	1 (20.0)	0 (0)
**Death (no, %)**	14 (9.2)	11 (8.9)	0 (0)	0 (0)	2 (40.0)	1 (16.7)

**Abbreviations**: AHA—autoimmune hemolytic anemia; COPD—chronic obstructive pulmonary disease; CNS—central nervous system; CRPD—chronic restrictive pulmonary disease; CVID—common variable immunodeficiency; ITP—idiopathic thrombocytopenic purpura; NRH—nodular regenerative hyperplasia (of liver); RTX—rituximab. ^∇^ Group B includes patients with a typical CVID clinical and laboratory phenotype, with a medical history of B-cell depletion immunotherapy for autoimmune or malignant disease several years ago (median: 9 years, range 6–14). ^ thyroid disease: hyper- or hypothyroidism; ^^ mono- or poly-arthritis (septic or reactive). ^#^ others included: vitiligo (7), pernicious anemia (5), fibromyalgia (4), eczema (3), alopecia (3), autoimmune neutropenia (4), autoimmune hepatitis/PBC (2), vasculitis (2), CNS myelitis (2), celiac-like disease (2), Sjogren-like syndrome (1), Raynaud syndrome (1). ^##^ enteropathy, intestine inflammation with follicular lymphoid hyperplasia, ^###^ allergic rhinitis, atopic dermatitis, allergic reactions to medicine (drugs, excluding IgRT). * including the development of nasal polyps (not only recurrent sinus infections), ** overactive spleenomegaly.

**Table 2:** Clinical and molecular data of patients with *CTLA4* mutations *.

Patient	Sex	Age Onset	Age at Diagnosis	Family History and Clinical Presentation	Molecular Defect	Further Complications and Outcome
#68	2	4	18	Proband. Recurrent upper respiratory infections, Crohn-like disease, autoimmune thyroiditis, pernicious anemia, tonsillar hypertrophy, sun-sensitive skin rash.	c.267C>A, p.Y89X	No (alive in good condition under IgRT)
#69	2	20	44	Mother of #68. Recurrent sinusitis and erycipelas, autoimmune thyroiditis, sun-sensitive skin rash.	c.267C>A, p.Y89X	No (alive in good condition under IgRT)
#116	2	25	28	Proband. Unknown family history. Recurrent CNS, upper and lower respiratory infections, nasal polyps, splenomegaly.	c.224G>A, p.R75Q	No (alive in good condition under IgRT)
#133	1	15	19	Proband. His father was a carrier of the same defect without any disease. Recurrent respiratory infections and autoimmune thyroiditis. Massive splenomegaly at diagnosis (diagnostic splenectomy).	c.267C>A, p.Y139C	Non-Hodgkin lymphoma 8 years after diagnosis (remission after treatment). Died 12 years after diagnosis due to a relapse of lymphoma
#134	2	7	7	Proband. Her father was a carrier of the same defect without any disease. Splenomegaly (diagnostic splenectomy), lymphadenopathy, followed several years later by recurrent lower respiratory infections, spontaneous peritonitis, severe granulomatous disease (liver, spleen, lungs)	c.208C>T, p.R70W	Cirrhosis, renal insufficiency, pulmonary hypertension. Died 14 years after diagnosis due an attack of spontaneous bacterial peritonitis

**Abbreviations**: CNS—central nervous system; IgRT—immunoglobulin replacement treatment. * Clinical and laboratory data for patients #68, #133, and #134, along with functional assays for the causative *CTLA4* defect, were also presented by us in previous manuscripts [16,17].

**Table 3:** Overview of laboratory findings of the patients of the study at diagnosis.

	Total	Group A (CVID)	Group B ^∇^ (“Secondary” Hypogamma)	Group C (c.IgAD and IgGsD)	Group D (*CLT4* Deficiency)	Group E (Unclassified Hypogamma)
No	153	123	12	7	5	6
**Immunoglobulin levels (mg/dL)**						
Total (mean± SDEV)	356.7 ± 265.1	306.0 ± 222.2	410.9 ± 281.2	894.7 ± 307.6	582.5 ± 88.0	472.5 ± 238.6
IgG (mean ± SDEV)	299.6 ± 231.0	255.5 ± 188.1	331.6 ± 206.5	833.6 ± 292.9	504.7 ± 112.0	362.2 ± 173.1
IgA (mean ± SDEV)	21.2 ± 42.6	16.2 ± 31.5	46.9 ± 91.6	9.4 ± 16.0	46.8 ± 13.2	70.0 ± 69.6
IgM (mean ± SDEV)	41.4 ± 61.6	36.9 ± 47.9	82.2 ± 150.8	51.8 ± 25.2	31.0 ± 29.2	40.3 ± 13.1
**IgG subclass levels (mg/dL)**	(90)	(65)	(10)	(7)	(2)	(6)
IgG1 (mean ± SDEV)	230.0 ± 177.2	185.6 ± 138.2	239.5 ± 172.6	561.4 ± 201.4	372.5 ± 84.1	254.3 ± 141.2
IgG2 (mean ± SDEV)	73.0 ± 71.3	61.3 ± 61.9	72.5 ± 56.3	164.0 ± 119.1	124.5 ± 26.2	75.3 ± 61.4
IgG3 (mean ± SDEV)	18.7 ± 19.9	15.7 ± 16.0	12.8 ± 9.7	49.6 ± 34.7	48.6 ± 7.9	14.1 ± 15.7
IgG4 (mean ± SDEV)	5.9 ± 14.6	6.7 ± 16.3	6.4 ± 12.8	0.9 ± 1.0	1.9 ± 1.1	4.1 ± 5.4
**Immunophenotyping data** (mean ± SDEV, range) ^	(114)	(89)	(7)	(7)	(5)	(6)
WBC (/mL)	7285 ± 2764, 2600–16000	7281 ± 2873, 3200–16000	7600 ± 3378, 2600–11400	8608 ± 1593, 6400–10900	5900 ± 2300, 3600–8200	6317 ± 2100, 3800–8700
Lymphocytes (/mL)	2102 ± 1547, 566–11281	2168 ± 1751, 825–11281	1921 ± 1148, 606–3805	2046 ± 655, 1264–3007	1810 ± 967, 566–2895	1712 ± 493, 1031–2270
T cells (/mL)	1610 ± 1180, 336–8585	1682 ± 1327, 536–8585	1350 ± 577, 551–3074	1635 ± 490, 1063–2293	1347 ± 770, 336–2200	1268 ± 453, 527–1866
T helper cells (/mL)	870 ± 628, 244–4828	869 ± 701, 244–4828	778 ± 296, 374–1235	994 ± 373, 655–1482	822 ± 401, 245–1124	888 ± 411, 365–1469
T cytotoxic cells (/mL)	717 ± 762, 60–6058	779 ± 850, 60–6058	660 ± 576, 168–1819	578 ± 124, 369–721	511 ± 494, 66–1213	379 ± 117, 240–540
NK cells (/mL)	228 ± 297, 10–2346	224 ± 326, 10–2346	247 ± 279, 45–824	200 ± 158, 29–454	230 ± 195, 56–407	274 ± 162, 100–476
B cells	195 ± 141, 0–811	198 ± 146, 5–811	156 ± 152, 0–431	173 ± 135, 37–412	232 ± 99, 137–335	161 ± 129, 29–405
Switched-memory (IgD^−^CD27^+^) (% of B cells)	4 ± 6, 0–26	3 ± 4, 0–12	1 ± 2, 0–3	5 ± 4, 0–10	1 ± 1, 0–2	10 ± 8, 1–26

**Abbreviations**: NK cells—natural killer cells; RTX—rituximab; WBC—white blood cell count. * Into brackets are the number of patients for whom IgG subclasses and immunophenotyping data were available at diagnosis. ^∇^ Group B includes patients with a typical CVID clinical and laboratory phenotype, with a medical history of B-cell depletion immunotherapy for autoimmune or malignant disease several years ago (median: 9 years, range 6–14). ^ The higher levels of leukocyte subpopulations were observed in a patient with CVID carrying a *TNFRSF13B*-p.C104R mutation, who subjected to splenectomy (due to a massive splenomegaly) 7 years before diagnosis; although even at that time the patient displayed hypogammaglobulinemia and granulomas formation in spleen, the diagnosis of CVID delayed for seven years! Respectively, the lower levels of leukocyte subpopulations correspond to patients receiving corticosteroid treatment for autoimmune manifestations when the initial immunophenotype analyses were performed.

## Data Availability

Data available on request on request from the corresponding author.

## Supplementary Materials

The following supporting information can be downloaded at: https://www.mdpi.com/article/10.3390/medicina60050782/s1, Table S1: Clinical and laboratory characteristics of patients developing severe hypogammaglobulinemia after rituximab treatment.
