# Supplementary material for: A Nationwide Study of the Delayed Diagnosis and the Clinical Manifestations of Predominantly Antibody Deficiencies and CTLA4-Mediated Immune Dysregulation Syndrome in Greece
Source: Medicina (Kaunas). 2024 May 8;60(5):782. doi: 10.3390/medicina60050782 (PMC11123397; doi:10.3390/medicina60050782)
Supplement: Supplementary file 1 [file medicina-60-00782-s001.zip › medicina-2908113-supplementary.pdf]

Supplementary Table 1. Clinical and laboratory characteristics of patients developing severe hypogammaglobulinemia after rituximab treatment.

No	Age	Sex	Initial diagnosis (age) and treatment	Age at diagnosis of immunodeficiency	Years after RTX treatment	Clinical and laboratory characteristics at CVID diagnosis	Years of follow-up
1	34	Male	Refractory Evans syndrome (at 3 years old) without hypogammaglobulinemia. Due to relapse of Evans syndrome, he received RTX when 19 years old. Medical history of bronchitis and pneumonia attacks before autoimmunity.	11	-	Recurrent upper and lower respiratory infections, splenomegaly/splenectomy, lymphadenopathy.<br>IgG 466 mg/dL, IgM 20.9 mg/dL, IgA 68.4 mg/dL	23
2	26	Male	Mature B-ALL (treated with chemotherapy including RTX) when he was 14 years old, without hypogammaglobulinemia	22	8	Recurrent urinary, upper, and lower respiratory infections. No response to PCP vaccine. Arthralgias<br>IgG, IgM, IgA almost undetectable levels	4
3	34	Male	Orbital DLBL non-Hodgkin lymphoma (19 years old) without hypogammaglobulinemia, receiving chemotherapy including RTX. Medical history of recurrent infections also in childhood	33	14	Recurrent upper respiratory, gastrointestinal, and skin infections. Eczema. No response to PCP vaccine.<br>IgG 563 mg/dL, IgM 35.0 mg/dL, IgA 0.0 mg/dL	1
4	61	Female	DLBL non-Hodgkin lymphoma without hypogammaglobulinemia (at 50 years old), receiving chemotherapy including RTX. History of Raynaud syndrome.	60	10	Recurrent upper and lower respiratory infections. No response to PCP vaccine.<br>IgG 445 mg/dL, IgM 0.0 mg/dL, IgA 301.0 mg/dL	1
5	38	Female	DLBL non-Hodgkin lymphoma (mediastinum) without hypogammaglobulinemia, receiving	33	8	Recurrent upper and lower respiratory infections.	5

			chemotherapy including RTX. Medical history of vitiligo, psoriasis, hypothyroidism.			IgG 291 mg/dL, IgM 18.9 mg/dL, IgA 38.7 mg/dL	
6	24	Male	Refractory Evans syndrome (at 10s years old) receiving RTX after splenectomy.	21	8	Myocarditis, lymphadenopathy, resistant autoimmune thrombocytopenia, recurrent upper and lower respiratory infections. No response to PCP vaccine. IgG 126 mg/dL, IgM 302 mg/dL, IgA 0.0 mg/dL	3
7	54	Male	Refractory autoimmune hemolytic anemia (at 39 years old) receiving RTX (thereafter he remained in complete remission for seven years). No immunoglobulin levels available at initial diagnosis.	46	7	Recurrent autoimmunity (thrombocytopenia, autoimmune hemolytic anemia – Evans syndrome), recurrent intestinal infections. The patient received several medicines for refractory Evans syndrome and was subjected to splenectomy. Currently in good condition with sustained hypogammaglobulinemia. IgG 183 mg/dL, IgM 23.7 mg/dL, IgA 0.0 mg/dL. *TNFRSF13B*-p.C104R	9
8	25	Male	Recurrent autoimmune thrombocytopenia without hypogammaglobulinemia (at 9 years old). RTX administration due to resistant disease.	15	6	Recurrent upper and lower respiratory infections, chronic restrictive pulmonary disease, granulomatous disease, splenomegaly, possible GLILD. IgG 554 mg/dL, IgM 6.0 mg/dL, IgA 148 mg/dL.	10
9	38	Male	Mature ALL when 20 years old, receiving chemotherapy including RTX.	31	11	Lymphadenopathy. Due to severe and sustained hypogammaglobulinemia (without severe infections), he receives IgRT (sustained hypogamma for 18 years) IgG 189 mg/dL, IgM 47.6 mg/dL, IgA 6.7 mg/dL.	7

10	49	Male	Marginal zone non-Hodgkin lymphoma at 34 years old, receiving chemotherapy including RTX. Relapse with DLBC lymphoma 2 years later (autologous BMT). Medical history of atopy.	46	12	Recurrent (and severe) upper and lower respiratory infections (recurrent hospitalizations). No response to PCP vaccines (undetectable IgM and IgA levels during follow-up and after IgRT).<br>IgG 295 mg/dL, IgM 0.0 mg/dL, IgA 0.0 mg/dL.	3
11	30	Male	Burkitt lymphoma at 18 years old (without hypogammaglobulinemia) receiving chemotherapy with RTX. Relapse with Hodgkin's lymphoma when 25 years old. Medical history of recurrent sinusitis when 10 years old, alopecia at 4 years old.	27	12	Recurrent (severe) upper respiratory infections (sinusitis). No response to PCP vaccine. Sustained hypogammaglobulinemia (undetectable IgM and IgA levels during follow-up and after IgRT)<br>IgG 192 mg/dL, IgM 0.0 mg/dL, IgA 0.0 mg/dL.	3
12	39	Female	Recurrent autoimmune thrombocytopenia and splenomegaly (at 27 years old, receiving RTX treatment) without hypogammaglobulinemia	36	9	Recurrent upper and lower respiratory infections, splenomegaly. No response to PCP vaccine.<br>IgG 475 mg/dL, IgM 104 mg/dL, IgA 0.0 mg/dL.	3

Abbreviations: ALL, acute lymphoblastic leukemia; BMT, bone marrow transplantation; DLBC, diffuse large B cell (lymphoma); GLILD Granulomatous and Lymphocytic Interstitial Lung Disease; IgRT, immunoglobulin replacement treatment; PCP, Pneumococcal conjugate polysaccharide (vaccine); RTX, rituximab
